# COVID-19 and the efficiency of health systems in Europe

**DOI:** 10.1186/s13561-022-00358-y

**Published:** 2022-02-12

**Authors:** Dan Lupu, Ramona Tiganasu

**Affiliations:** Alexandru Ioan Cuza University of Iasi, 700483 Carol I Boulevard, 22, Iasi Romania

**Keywords:** COVID-19, European health systems, Efficiency, Data envelopment analysis (DEA), Policy recommendations, C14, C61, C67, H51, I18

## Abstract

**Background:**

This study aims at analyzing the efficiency of the health systems of 31 European countries in treating COVID-19, for the period January 1, 2020 – January 1, 2021, by incorporating some factors from a multidimensional perspective.

**Methods:**

The methodology used in the research was Data Envelopment Analysis (DEA), through which efficiency scores for health systems have been calculated. The research was performed considering three stages: the first wave (January 1–June 15), the relaxation period (June 15–October 1) and the second wave (October 1–December 31). In evaluating the determinants of the efficiency of health systems, six major fields of influence were taken into account: health care, health status, population, economic, cultural/societal and governmental issues, all covering 15 indicators. After measuring the efficiency, we used the Tobit type regression to establish the influencing elements on it.

**Results:**

The results for the public health systems of European states were determined for each country and period. We evaluated the efficiency of health systems in Europe against COVID-19, starting from health inputs (COVID-19 cases, physicians, nurses, hospital beds, health expenditure) and output (COVID-19 deaths). The obtained outputs show that, especially in the first phase of the pandemic, the inefficiency of the health systems was quite high, mainly in Western countries (Italy, Belgium, Spain, UK). In the relaxation phase and in the second wave, the Western states, severely affected at the beginning of the pandemic, began to take adequate measures and improve the efficiency of their sanitary systems. Instead, Eastern European countries were hit hard by the inefficiency of health systems (Bulgaria, Greece, Hungary, Romania). After Tobit regression, results of the study show that the influencing elements are different for the three stages: concerning the first wave, comobirdities, population age, and population density are important; for relaxation period a great influence have government effectiveness and power distance; with respect to second wave, the relevant factors are education and population density.

**Conclusions:**

The results obtained could serve as starting points for health policymakers to perform comparative analyzes in terms of good practices in the health system and to develop national plans to better deal with health crises. At the same time, they can be used internationally to achieve a coherent and effective community response to the pandemic.

## Introduction

The relevance of approaching a topic revolving around COVID-19 lies in the multitude of its negative implications, felt at all levels of society. In combating them, a major role is played by the type of crisis management that applies in each country. Although, over time, Europe has faced various crises, which have varied in intensity, the COVID-19 pandemic has perhaps one of the most pronounced dynamism related to the rapid transmission of the virus and the loss of human lives. The globalized context of this crisis requires that, periodically, inventories be made in terms of the input-output relationship, so that the way of action of decision makers and the adaptation of governance systems can be done in accordance with the particularities of each state. The quality of the responses provided to the crisis depends, in particular, on endogenous factors. Furthermore, path development evolution significantly affects the ability to react to shocks. Theoretically, if in the case of other health crises (SARS, MERS-COV, bird flu, Ebola) some countries might not have been prepared to face such challenges, in practice, the experience gained by going through them, should lead to the implementation of transformative policies to stabilize systems. Even if each crisis is accompanied by uncertainties, a risk prevention strategy should exist a priori, the effectiveness of measures being closely linked to the ability of governments and of those with decision-making power to anticipate possible shocks that may arise [1]. The complexity and accelerated diffusion rendered this crisis to have multidimensional valences, forcing governments to have immediate reactions, without which the consequences of such a disruption of the global system could have the worst repercussions, in terms of people dead or left with sequelae, especially in the respiratory tract.

After its outburst in China in December 2019, COVID-19 began to spread rapidly around the world, in just a few months reaching all countries; in Europe, it reached a large scale spread in the spring of 2020. Although Western European countries benefit from high-performance health systems, the COVID-19 crisis has severely brought Italy, Spain, France and the UK to the forefront of the crisis: Spain (566326), United Kingdom (3651789), France (353986), Italy (286297) and Germany (259428). Eastern European countries, with weaker health systems, coped better with this crisis, in the first phase. Against this background, the legitimate question is how this overwhelming difference in the efficiency of health systems occurred in the first wave of the crisis. However, in the relaxation period, and in the second wave, the roles change: the Western states with better sanitary systems manage to respond more adequately to the crisis; instead, the Eastern states are undergoing dramatic negative changes. Therefore, if at the beginning of the pandemic there was a stronger destabilization in terms of health of Western European economies compared to those in the East. Later, starting with the summer of 2020, it turned out that states that demonstrated prompt action capabilities, adapted context, were those who managed to outline the premises of a considerable decrease in the number of infections. In addition, the relationship between resources and needs has been streamlined, in order to minimize the risk of blockages in intensive care units, in terms of the number of existing beds or specialized medical staff.

Looking back to 2020, it can be said in unison that both governments and the world’s population have been subjected to severe trials in the face of the health crisis and its consequences. In the context of increased uncertainty, governments have had to adapt to the health, economic and social challenges posed by the pandemic, the latter showing even more accurately the existing problems of medical systems, amplifying their shortcomings. In essence, the differences between public health systems are determined by infrastructure, the quality of public policies, culture, legislation, human resources; at European level, there are constant concerns for finding solutions to support reducing these discrepancies. A shock as intense as that created by the coronavirus has caused states with advanced health systems to encounter serious difficulties in controlling the health consequences of COVID-19. The resources of the health system (doctors, nurses, beds) were insufficient at the level of European states, most of them reaching the maximum capacity of the intensive care units. Despite all the financial and technical allocations, the ability to cope with this shock was really challenging, given the rapid development of the pandemic and the huge needs generated. With regard to the Member States, the EU cannot intervene directly or impose binding decisions, as public health is a common task shared between them and the EU. At the time of the first coronavirus cases, Member States applied their own health measures, and as a guide to good practice, the European Centre for Disease Control (ECDC) made a number of general recommendations for pandemics (quarantine, social distancing) or specific ones (risk assessment for the population, increased communication, support for vulnerable people).

The assessment of the strict measures with which the governments of the EU countries managed the pandemic during 2020 can be accounted based on the Stringency Index, developed by the University of Oxford [2]. This index consists of nine response indicators, quantified on a scale between 0 and 100, where 100 represents the maximum strictness, and refers to strategic and control measures such as: school closure; prohibiting the travel of those who practice jobs that can be done from home; cancellation of public events; restrictions on public meetings; cancellation of trips by public transport; home stay requirements; public information campaigns; restrictions on internal movements; and international travel controls. It should be noted that a higher score does not necessarily mean that a country’s response is better compared to others that have scored lower, as this index does not assess the effectiveness of government policies. The differences for the year 2020 in terms of this index between the countries integrated in the EU after 2004 and the old member states are captured in Appendix 1*.*

Although the harshness of the measures could be perceived by the population as a violation of fundamental freedoms, it is certain that this is a situation that endangers people’s health or even their lives, and safety and the provision of a minimum level of protection should take precedence over the existence of scientifically unfounded opinions. Over time, medical research specialists have made considerable efforts to find different treatments for many diseases, the results identified being validated by control bodies (World Health Organization, European Medicines Agency – EMA, etc.) and therefore the role of national governments and in this case the EU, as a regional and global player, should also be to strengthen public confidence in the demonstrated effectiveness of the anti-COVID vaccine, administered since the end of 2020. This can be achieved through extensive awareness campaigns, leaving no room for false news to diminish the merits of science to create beneficial effects on a large scale. In addition, the recommendations regarding the protection conferred by observing the hygiene rules should not be neglected because they can mean the protection of those close to them and their right to life. There are studies that demonstrate the direct link between the level of education and the openness to the acceptability of health safety measures [3, 4].

The analysis of the efficiency of health systems in pandemic management was performed for 2020, considering the three periods of development: first wave (January 1–June 15), relaxation (June 15–October 1) and second wave (October 1–December 31). The variables used in the present research to calculate the efficiency of European countries to COVID-19 are selected based on a complex approach and consequently, an appropriate technique is needed to manage multiple inputs and outputs associated with the topic. Thus, we resorted to Data envelopment analysis (DEA), which is a technique used to measure the performance of health systems that can manage multiple input/output variables. In this study, the DEA method analyses the entry parameters (number of diseases, doctors, nurses, health expenditure per capita) and exit ones (number of deaths) and objectively establishes the efficiency of the European health systems. The efficiency scores obtained after applying this method show the performance of each European state in relation to other countries for that period. The effective response to the pandemic in the three phases of 2020 meant minimizing the death rate and maximizing the number of patients treated. Therefore, this paper aims to identify the main factors that have an impact on the trend of deaths recorded, at country level, from January to December 2020, for 31 European countries. The main hypothesis of the research is to see if the efficiency of European health systems is determined not necessarily by factors related to the health system, but also by economic, social, governmental characteristics. Research on this topic is in its infancy, so our study adds to the limited literature by analysing several groups of factors at country level, taking into account almost all European countries. The novelty of the study is given by the temporal analysis of the efficiency of the European public health systems, separately on the three phases corresponding to 2020, by incorporating some factors from a multidimensional perspective.

## Literature review

The evaluation of the performance of the health systems represents an intensely debated topic at the level of international organizations, the governments and the population, as it has direct implications on each citizen. Therefore, the goal of generating efficiency in any health sector should be the main concern when designing development strategies and plans. When measuring health efficiency, direct reference should include, on the one hand, technical efficiency, in order to obtain more outputs compared to inputs, and on the other hand, allocative efficiency, which means that the distribution of resources is done rationally so that the highest outcomes occur at the lowest costs [5]. In relation to these aspects, we considered it interesting to see which countries have a higher efficiency of health systems: those that have adopted the Beveridge model (Cyprus, Denmark, Spain, Finland, Ireland, Italy, Latvia, Malta, Portugal, United Kingdom, Sweden), which is based on a predominant tax financing (National Health System), or those that are framed in the Bismarck model (Germany Austria Belgium Bulgaria Croatia Slovakia Slovenia Estonia France Greece Hungary Lithuania Luxembourg Netherlands Poland Czech Republic Romania), which implies that the financing of the health system is made through compulsory social security contributions, usually by employers and employees (Social Health Insurance System). In addition to these two models, there is also the mixed model, in which private financing from voluntary insurance systems is significant (Private Health Insurance System).

The literature contains studies that analyse the efficiency of public health systems, mainly at the microeconomic level, i.e., patient, hospitals, measures and health programs [6, 7], as well as comparative research at the macro level, between states or regions [8, 9] however, the latter are much fewer than the former. Efficiency in the health sector needs to be debated by experts in order to identify drivers, respectively obstacles, in the way of health systems. A specific method used to quantify the efficiency of health systems refers to data envelopment analysis (DEA), which has been used in various research, but, to our knowledge, to calculate the efficiency of European health systems during a pandemic and especially of COVID-19, this method has not been properly included in the studies, as mainly parametric/ nonparametric analyses (stochastic frontier analysis - SFA) have been used.

The performance of the public health system is calculated in these studies based on general indicators related to resources (physicians, nurses, beds, health expenditures) and outcomes (transposed in quality of life). We summarize, briefly, some results of these studies:

- by analyzing 34 OECD countries from the perspective of health systems efficiency, using 14 inputs (pharmaceutical consumption, average years of schooling, obesity, tobacco consumption, alcohol consumption, per capita health expenditure, percentage of health care expenditure, physicians, nurses, beds) and 4 outputs (life expectancy, infant mortality, population aged and population aged 65 years and older), it is concluded that there are substantial differences between countries’ health systems and subsystems, which require specific approaches to improve their performance [7];
- applying the analysis to the level of 29 OECD countries, in the period 2000–2010, it is highlighted that a positive influence on the efficiency of health systems is given by education, income, environment, and a negative one by health expenditures, public or private [10];
- at the level of 34 OECD countries, the DEA method is applied to rank the states in terms of the efficiency of health systems and a strong efficiency is obtained in Norway, Sweden, Germany, South Korea, Singapore, Japan, while the other states present lower efficiencies [8];
- studying the health systems for 31 OECD countries taking into account both health system variables (doctors, beds and health expenditure) and external ones (GDP, institutional arrangements, population behavior, socio-economic or environmental determinants), the conclusion was that external factors are the ones that have a greater influence on efficiency than health factors [6];
- including in the study 32 OECD countries, it is highlighted that a number of factors (obesity, smoking, low GDP per capita and education level) negatively influence the efficiency of health systems, while environmental factors have a positive influence [11].
- by developing a theoretical model for establishing resource allocation decisions that affect multiple beneficiaries, it is shown that for maximizing efficiency it is necessary to consider the fairness, and with reference to medical decisions made by the World Health Organization (WHO), they should to be done at a certain aggregate level, rather than separately for each affected individual [12].

Although studies on the impact of macro factors on health systems during the pandemic are very few, we present in the following some of their conclusions:

in order to calculate the efficiency of the health systems of 16 countries, in the first 5 weeks after the pandemic, it is shown that most states have experienced a decrease in efficiency, while some (South Korea, Singapore, China) have managed to adopt appropriate measures to limit the virus [13];

analyzing 543 micro-regions in Brazil, it is concluded that richer regions, although more severely affected by the pandemic, were able to treat and reallocate patients better than poor regions, thus showing that socio-economic inequalities have an important impact in this health crisis [9];

regarding the efficiency of the health system in Malaysia, it is estimated that it was mainly determined by medical care process, strong response and preparedness to pandemic, rapid allocation of resources and less by social measures to limit the virus [14];

in the spatial analysis to study the spread of the pandemic in 123 European regions in 12 countries, it is shown that a number of factors (GDP per capita, unemployment rate, share of aging population) negatively influence the phenomenon, instead of other factors (number of doctors, beds) have a positive influence [15];

studying three types of measures taken (global travel ban, national school closure and national lockdown) of 130 countries, using Google’s social mobility reports, it is highlighted that a series of characteristics of states (health preparedness, higher GDP per capita, higher democracy political systems, larger population) led to a delay in the implementation of strict health policies [16];

in the fight against coronavirus and implicitly in ensuring a high efficiency of health systems a special influence have the institutional and economic factors, as well as the allocation of resources, coherence of measures and behavior of individuals [16–18] in addition, the example of New Zealand is brought to the fore, because through its government responses to the COVID-19 crisis has managed to impose appropriate policies (lockdown, stimulus package, and travel ban) to limit the spread of the virus, which, finally, led to some stock market stability [19].

The diversity of the results given by the literature shows the incipient nature of the research on this topic. When analyzing data at the country level, the influencing factors found significant in explaining the variation of cases of infection or death cover a variety of areas. As such, we will perform our analysis taking into account the indicators in health, demography, social, cultural, institutional and economic areas, using logit and quantile regressions for each category, to capture as many effects as possible from all these determinants.

## Data and methodology

The sample of countries comprises thirty one European countries, namely: EU members states, Iceland, Norway, Switzerland, UK. A diversity of sources was used for collecting the data: Worldometer, World Health Organization and World Bank. The data regarding the death rates caused by COVID-19 were collected from Worldometer, the health-related data were extracted from WHO, and the socio-economic data from World Bank, all at country-level. The COVID-19 data covers the period January–December 2020 and the data referring to health and socio-economic indicators were collected for the year 2019 and are presented in detail in Table 1*.*

**Table 1:** List of determinants of the COVID–19 death rates

	Explanatory variable	Definition	Data source	References
INPUT	COVID-19 cases	COVID-19 cases per 1 million population	WHO	[9, 13–16, 18, 20–23]
	Physicians	Physicians (per 1000 population)	WHO/ Eurostat	[6–11, 14–16, 18, 20, 22, 24–27]
	Nurses	Nurses and midwives (per 1000 people)	WHO/ Eurostat	[7–11, 15, 18, 20, 24–26]
	Hospital beds	Hospital beds (per 1000 population)	WHO/ Eurostat	[8–11, 14, 15, 18, 20–22, 24–27]
	Health expenditure	Current health expenditure per capita (current US$)	WHO/ Eurostat	[8, 9, 11, 14, 18, 20–22, 24–27]
OUTPUT	COVID-19 deaths		WHO	[9, 14–16, 18–23]
driving factors for efficiency	Comorbidities	Probability (%) of dying between age 30 and exact age 70 from any of cardiovascular disease, cancer, diabetes, or chronic respiratory disease	WHO	[7, 8, 18, 20, 21, 25, 26]
	Population over 65	Population ages 65 and above in the total population (%)	World Bank	[8, 15, 18, 20, 21]
	Population density	People per sq. km	World Bank	[9, 15, 18, 21, 28, 29]
	Education	Percentage of population with intermediate level of education	World Bank	[6, 8, 11, 21, 22, 24, 26]
	GDP per capita	GDP per capita (constant 2010 $)	World Bank	[8–11, 15, 18–22, 24–27]
	Power distance	This dimension expresses how a society handles inequalities among people*	Hofstede insight	[14, 16, 21, 28–30]
	Government Effectiveness	Perceptions of the quality of public services	World Bank	[7, 9–11, 14, 22, 24, 26, 28–30]

* People in societies exhibiting a large degree of power distance accept a hierarchical order in which everybody has a place and which needs no further justification. In societies with low power distance, people strive to equalise the distribution of power and demand justification for inequalities of power

The factors that could explain the variations of the mortality rate between countries are numerous and different, the studies on this topic not being outlined in a comprehensible way, which would capture the particularities of each state, in order to select relevant information. Thus, we used in our analysis four categories of indicators: health, demographic, economic and cultural/societal, with a total of 15 variables. For the econometric analysis, we assessed the health status of individuals based on comorbidities. The relevance of studying the effects of health infrastructure is given by the fact that the increased performance of a country’s health system can have a positive impact on the effectiveness of the authorities’ response to COVID-19 infection and death. Then, the demographic structure could be an influential factor in the analysis of mortality rates, because an elderly population has more health problems and higher probabilities of death in a health crisis. Thus, we considered the proportion of the population over 65 and the age dependency ratio as variables to assess the size of vulnerable groups of the population, both in terms of exposure and response to the virus and of negative consequences on the health system. Population density measures congestion and mobility, as this is an important factor in controlling the rate of contamination. Referring to economic determinants, they were selected to evaluate the potential of the economy to have significant interventions in the event of a pandemic. We used GDP/capita to measure the size of the economy, and education (tertiary) to assess the economic disadvantages of individuals in a country, because this might turn out to be a potential determinant of infectious diseases. Societal and cultural determinants measure a society’s progress in terms of individual behaviors and beliefs (power distance), and a government’s social responsibility to increase the quality of life of its citizens (government effectiveness). Culture is a corruption-health nexus, affecting the physical and mental health of the population (a high level of corruption affects more deeply the physical health of people in low-income countries than in high-income countries, and mental health is more strongly influenced by corruption in high-income countries) [30]. All the data is transformed using logarithm.

### Methodology

Data Envelopment Analysis (DEA) is a non-parametric mathematical method developed by [31, 32]. Based on linear programming, the method measures the productive efficiency of a set of Decision Making Unit (DMU) by empirically constructing an efficiency frontier. DEA methodology is extremely flexible and has been widely used in calculating efficiency in various fields. DEA starts from a series of variables, input and output, and calculates the efficiency of each DMU, elaborating a ranking of relative efficiency with attribution of weights. The efficiency frontier is made up of efficient DMUs, located on it, compared to the inefficient ones (the distance from the DMU to the border represents efficiency) [33].

Efficiency is measured by solving a linear programming problem for each DMU_i_:

Max ${\sum}_{i=1}^m uiyi$ with the following conditions:

$$ {\sum}_{j=1}^n vj\  xj0 $$=1.

$$ {\sum}_{i=1}^m ui\  yik $$ - $$ {\sum}_{j=1}^n vj\  xjk $$ ≤ 0, for k = 1,2 … h and u_im_ > 0, v_in_ > 0.

The efficiency of a DMU_i_ is calculated as the ratio between weighted sum of outputs and to a weighted sum of inputs [34]: (${\sum}_{i=1}^m uiyi$ / ${\sum}_{j=1}^n vj\  xjk$).

The inputs and outputs for the model are presented in Table 1. After calculating the efficiency for each state, we will use the Tobit type regression to identify the influencing factors on them. The robustness of the analysis will be tested by quantile regresion.

## Results

Table 2 shows the descriptive statistics for the data from our study, whose source is WHO, World Bank, Eurostat, and Worldometers. They show mean, maximum, minimum and standard deviation. In the first wave, the average number of COVID-19 cases in European countries was 51,065, with a minimum of 671 in Malta, a maximum of 286,227 in the UK, and a standard deviation of 83,266. The number of deaths in the first wave had an average of 5727 people, with a minimum of 9 people in Malta, a maximum of 40,613 in the UK and a standard deviation of 11,373. During relaxation, the average number of cases was 67,918, with a minimum of 809 in Cyprus, a maximum of 577,457 in Spain, and a standard deviation of 131,582. The number of deaths during the relaxation period averaged 592 people, with a minimum of 0 people in Iceland, and a maximum of 4797 people in Spain, and a standard deviation of 1082. The second wave of the pandemic in Europe led to an average number of 491,193 infections, with a minimum of 2882 in Iceland, a maximum of 2,172,019 in the UK, and a standard deviation of 622,736. The number of deaths caused by the pandemic had an average of 8633 people, with a minimum of 19 people in Iceland, a maximum of 39,017 in Italy, and a standard deviation of 11,108. Therefore, as a whole, there is a worsening of the situation in the second wave, due to the rather high contagiousness of the virus and the lifting of some restrictions during the relaxation period (June 15–October 1), which overlapped and with the performance of summer vacations, when people did not exactly follow the rules of social distance, wearing a mask, etc.

**Table 2:** Descriptive statistics of the input and output variables used in this study

Variables	Mean	Maximum	Minimum	Std. Dev.
Cases_first wave period	51,065.52	286,227.0	671.00	83,266.24
Deaths_first wave period	5727.64	40,613.00	9.00	11,373.38
Cases_relaxation period	67,918.84	577,457.0	809.00	131,582.2
Deaths_relaxation period	592.87	4797.000	0.000	1082.33
Cases_second wave period	491,193.9	2,172,019.	2882.000	622,736.3
Deaths_second wave period	8.633	39,017.00	19	11,108.29
Physicians per 1000 people	3.41	5.21	2.18	0.78
Nurses and midwives per 1000 people	9.96	19.46	3.63	4.31
Hospital beds per 1000 people	5.01	8.30	2.60	1.61
Current health expenditure (per capita current US$)	3331.24	9956.26	555.10	2395.18
Probability of dying between age 30 and exact age 70 from any of cardiovascular disease, cancer, diabetes or chronic respiratory disease	14.24	24.60	8.60	5.11
Population ages 65 and above (of total population)	18.81	22.75	13.71	2.48
Population density (people per Sq km of land area)	174.77	1514.46	3.51	272.04
GDP per capita (current US$)	40,239.26	116,597.3	9271.54	25,746.51
Government effectiveness	82.90	99.51	43.26	12.80
Power distance	49.70	100.00	11.00	20.32
Tertiary education	32.18	42.80	16.20	7.39

Summarizing the results regarding the other variables included in the analysis, the following were found: *the average number of doctors per 1000 people* in Europe was 3.41, with a minimum of 2.18 in the Netherlands and a maximum of 5.21 in Greece. The number of nurses per 1000 inhabitants knows significant differences: a European average of 9.96 people, but with a double maximum of 19.46 in Belgium and a minimum three times lower, of 3.63 in Greece. Then, the indicator related to *the number of hospital beds* shows major differences: a European average of 5.01, but with a maximum almost double (8.30) in Germany and a minimum of half (2.60) in Sweden. *Health expenditure* has significant individualizations between European countries: an average of 3331.24, but with a triple maximum (9956.26) in Switzerland and a minimum 6 times lower (555.10) in Romania. The variable that refers to *comorbidities* records major differences between European countries: an average of 14.24% people with serious diseases, with a double maximum of 24.60 in Malta and a minimum of 8.60 in Switzerland. *The share of the elderly population* shows large individualizations in Europe: an average of 18.81, with a maximum of 22.75 in Italy and a minimum of 13.71 in the Czech Republic. However, *the population density* has the highest diversifications at European level: an average of 174.77, a maximum 10 times higher (1514.46) in Malta, and a minimum 50 times lower (3.51) in Iceland. *Government effectiveness* at European level has a high average of 82.90, with a maximum of 99.51 in Switzerland, but also a minimum of half (43.26) in Romania. *Tertiary education* has an average of 32.18 of the European population, with a maximum of 42.80 in Ireland, and a minimum of 16.20 in Romania.

DEA methodology makes it necessary to perform a correlation test between the input and output variables to determine how to influence them [32], the coefficients obtained separately for the 3 phases of the pandemic being shown in Table 3. The number of cases and deaths exceeds 0.94 for all phases, which shows a high conditionality between them. There are also strong correlations between health expenses and the number of doctors. All variables are associated with a significance level of 0.01, which makes them usable in the DEA analysis [35, 36].

**Table 3:** Correlation test for input-output variables

	First wave period	Relaxation period	Second wave period
	(deaths)	(deaths)	(deaths)
deaths	1	1	1
cases	0.973	0.940	0.967
beds	0.214	0.040	0.143
health expenditure	0.107	0.319	0.276
physicians	0.403	0.373	0.340
nurses_	0.072	0.132	0.081

The DEA results were obtained using DEAP 2.1/ Octave software [35] and are presented in Table 4. For *the period of the first wave*, it can be seen that 19 European countries have the value of 1, i.e., they were effective in combating the pandemic; most of them are Eastern European or the small states that were able to quickly introduce a total lockdown. The states with the lowest efficiency values ​​(0–0.25) in this period are Belgium, Italy, UK, which also had the highest number of deaths. In the range of 0.25–0.50 efficiency there is the Netherlands, Spain; in 0.50–0.75 there are Romania, Switzerland, Sweden, Germany, and in 0.75–1, the rest of the countries are included. *Regarding the relaxation period*, it can be seen that 18 countries had efficiency (58%), recording the value of 1; Italy, Poland < 0.75; Sweden, Greece < 0.50; Bulgaria, Romania < 0.25; the other countries < 1. For *the period of second wave*, only one country (Norway) is efficient (3%), the rest of the countries having, in general, lower efficiencies than in the first wave. The results obtained are similar to the previous ones obtained by [13, 14, 17, 18, 20], which in turn calculates the efficiency of some components of the health system during the COVID-19 pandemic.

**Table 4:** COVID-19 and the efficiency of health systems in Europe

Country	First wave period	Relaxation period	Second wave period	Mean	Country	First wave period	Relaxation period	Second wave period	Mean
Austria	1	1	0.542	0.847	Italy	0.222	0.638	0.429	0.429
Belgium	0.166	0.842	0.498	0.502	Latvia	1	1	0.58	0.86
Bulgaria	1	0.175	0.724	0.633	Lithuania	1	1	0.658	0.886
Croatia	1	1	0.486	0.828	Luxembourg	1	1	0.736	0.912
Cyprus	1	1	0.868	0.956	Malta	1	1	0.504	0.834
Czechia	1	0.86	0.546	0.802	Netherlands	0.346	0.887	0.801	0.678
Denmark	1	1	0.867	0.955	Norway	1	1	1	1
Estonia	1	1	0.815	0.938	Poland	0.771	0.538	0.422	0.577
Finland	1	1	0.782	0.927	Portugal	0.801	1	0.611	0.804
France	0.782	0.879	0.561	0.74	Romania	0.669	0.146	0.356	0.39
Germany	0.752	0.867	0.551	0.723	Slovakia	1	1	0.665	0.888
Greece	1	0.324	0.474	0.599	Slovenia	1	1	0.347	0.782
Hungary	1	1	0.189	0.729	Spain	0.377	0.78	0.546	0.567
Iceland	1	1	0.824	0.941	Sweden	0.572	0.401	0.702	0.558
Ireland	1	1	0.801	0.933	Switzerland	0.71	1	0.612	0.774
					UK	0.235	0.804	0.605	0.548

Based on the DEA results, and especially on mean results (Table 4) the health systems of European countries could be grouped into three categories of efficiency [11, 18, 27]: *category 1*: high efficiency (0.75–1): Austria, Croatia, Cyprus, Czechia, Denmark, Estonia, Finland, Iceland, Ireland, Latvia, Luxembourg, Malta, Norway (only country with very high efficiency (= 1), Portugal, Slovakia, Slovenia, Switzerland; *category 2*: average efficiency: (0.5–0.75): Belgium, Bulgaria, France, Germany, Greece, Hungary, Netherlands, Poland, Spain, Sweden, UK’ category 3: low efficiency (< 0.5): Italy and Romania. States with low efficiency health systems have demonstrated vulnerabilities in how resources were used in the first year of the pandemic, by reference to the needs.

We have divided the DEA outputs into the four mentioned categories starting from the premise that several intervals on a [0–1] scale will allow us to better capture the differences between the health systems of the countries analyzed. Thresholds have been determined considering the response to the pandemic, the aim being to more clearly distinguish the group of states that are very efficient in managing the crisis from that of those that still have much to recover in this direction.

The results of the efficiency analysis are reported in Table 5. The average technical efficiency score was 83.3%, showing that most of countries are included in the sample operated close to the efficiency frontier. Of the 31 states analyzed, 20 or 64.5% were effective in the first wave of COVID-19 by imposing blocking measures, performing tests, using available doctors and predominant levels of health expenditure on gross domestic product (GDP). In other words, these 20 countries minimized (optimized) the use of all inputs in the model or used the inputs effectively in an attempt to be effective. The most inefficient countries were Belgium, Italy and UK (< 0.25), followed by Spain, Netherlands (0.25–0.50); during the relaxation period, the lowest efficiency of health systems was registered in Bulgaria and Romania, and in the second wave, in Hungary. Our results are similar to those obtained by [13, 17, 20].

**Table 5:** Results of the efficiency analysis

	First wave period	Relaxation period	Second wave period
*Intervals*	*Countries*	*Countries*	*Countries*
0.75–1	Rest of the countries	Rest of the countries	Rest of the countries
0.50–0.75	Romania, Swirtzerland, Sweden, Germany,	Italy, Poland	Austria, Bulgaria, Czechia, France, Germany, Latvia, Lithuania, Luxemburg, Malta, Portugal, Slovakia, Spain
0.25–0.50	Netherlands, Spain	Sweden, Greece	Belgium, Croatia, Greece, Italy, Poland, Romania, Slovenia
0–0.25	Belgium, Italy, UK	Bulgaria, Romania	Hungary

### Econometric analysis

To determine the drivers of health system efficiency, the present study will use Tobit regression analysis. Table 6 shows the results of the impact of independent variables (comobirdities, population age, population density, GDP per capita, education, government effectiveness, power distance) on the efficiency score (dependent variable).

**Table 6:** Determinants of health efficiency systems (coefficient and probability)

	Indicator	First wave period	Relaxation period	Second wave period
Health status	comobirdities	**−0.747 (0.015)**	− 0.453 (0.278)	0.335 (0.254)
	population age	**−0.766 (0.035)**	−0.574 (0.396)	− 0.244 (0.599)
	population density	**−0.246 (0.012)**	−0.010 (0.892)	**− 0.129 (0.012)**
Economy	GDP per capita	−0.207 (0.623)	0.049 (0.886)	0.050 (0.824)
	education	−0.525 (0.377)	−0.544 (0.261)	**0.601 (0.022)**
Government	government effectiveness	1.486 (0.177)	**3.433 (0.001)**	0.531 (0.351)
Cultural tradition	power distance	−0.187 (0.539)	**−0.070 (0.007)**	− 0.096 (0.603)
	Constant	0.626 (0.842)	−4.565 (0.076)	−1.117 (0.560)

For *the first wave*, the efficiency of health systems is negatively associated with comobirdities, population age, and population density, and this is due to the fact that, as a rule, countries with a larger aged population are associated with chronic health problems, making it difficult to fight against coronavirus. The other independent variables do not influence the efficiency score, having a significance threshold higher than 0.05. The results obtained are similar to those of [9, 15, 18, 20, 21, 25, 29] which showed that the effects of COVID-19 were stronger among the elderly and sick population.

For *the relaxation phase*, government effectiveness is positively associated with the efficiency score, this emphasizing, once again, the relevance of making appropriate decisions in the moments that require such actions; instead, it seems that cultural factors such as power distance negatively influence the efficiency of health systems, which means that there are variations in compliance with safety measures depending on inequalities in society (related to education, income, etc.). Our results are similar to those obtained by [9, 14, 16, 28, 29], for which show that the prompt and effective intervention of governments and the adherence of the population to these measures were decisive factors in combating COVID-19.

This last finding comes to complete, in *the second wave*, what was outlined in the relation period, namely that education influences the efficiency score, there is, therefore, a tendency for the population that is better and more correctly informed to comply with health measures; similar to the first wave, population density negatively influences the efficiency score.

The results obtained are similar to those obtained by [21, 22, 25, 37].

### Robustness test quantile regression

The quantile regression results are presented in Table 7 by quartile classes: 0.25, 0.50 and 0.75. Estimates show results similar to those obtained previously for Tobit-type regression. Even if there are still small differences in terms of coefficients, they are the same in terms of sign. For *the first wave*, comobirdities have a negative influence on the efficiency score at 0.5 quartile; population age also has a negative association at quartile 0.5, and population density has a negative influence at quartile 0.25. For *the relaxation perio*d, government effectiveness shows a positive association with efficiency at quartile 0.5, and power distance determines a negative influence on the efficiency score. For *the second wave*, population density negatively impacts the health efficiency score, and education has a positive association with it. The results validate the previously obtained estimates, which show that they are robust.

**Table 7:** Quantile regression estimates (coefficient and probability)

Indicator	First wave period			Relaxation period			Second wave period		
	0.25	0.50	0.75	0.25	0.50	0.75	0.25	0.50	0.75
comobirdities	1.231 (0.341)	**−0.579 (0.027)**	− 0.088 (1.000)	− 0.314 (0.743)	0.438 (0.471)	0.405 (0.433)	0.533 (0.188)	0.292 (0.452)	0.445 (0.334)
population age	0.598 (0.786)	**−1.327 (0.003)**	− 1.115 (1.000)	− 0.716 (0.499)	− 0.709 (0.448)	− 1.027 (0.309)	0.154 (0.820)	− 0.345 (0.548)	− 0.618 (0.411)
population density	**− 0.446 (0.012)**	− 0.044 (0.811)	− 0.037 (1.000)	− 0.018 (0.883)	0.009 (0.920)	0.006 (0.934)	**− 0.146 (0.044)**	− 0.111 (0.169)	−0.193 (0.102)
GDP per capita	−0.196 (0.847)	− 0.262 (0.718)	0.666 (1.000)	0.058 (0.914)	− 0.266 (0.563)	− 0.273 (0.540)	0.534 (0.116)	0.197 (0.569)	0.061 (0.888)
education	−1.589 (0.188)	−0.187 (0.884)	0.551 (1.000)	−0.376 (0.674)	−0.462 (0.484)	− 0.434 (0.465)	0.051 (0.927)	**0.309 (0.046)**	−0.221 (0.685)
government effectiv.	2.808 (0.159)	1.593 (0.433)	−5.335 (1.000)	2.209 (0.119)	**3.713 (0.002)**	3.294 (0.086)	0.303 (0.748)	0.591 (0.458)	1.482 (0.077)
power distance	−0.071 (0.915)	−0.238 (0.602)	0.136 (1.000)	−0.037 (0.917)	**− 0.006 (0.005)**	0.007 (0.073)	0.110 (0.624)	0.085 (0.733)	−0.168 (0.731)

## Discussion

The aim of this research was to investigate the efficiency of health systems in Europe and to identify possible determinants for them in time of COVID-19 pandemic. Based on the DEA methodology, it was possible to calculate the efficiency scores for the health systems for the three periods of the first year of the pandemic: first wave, relaxation and second wave. More importantly, after the initial analysis, we managed to individualize a series of characteristic of public health systems for these three periods. More precisely, our study included two methodological stages of analysis: the first stage consisted in estimating the efficiency of public health systems for European countries during the first year of the COVID-19 pandemic separately for three subperiods, using the DEA methodology and the states as decision-making units (DMU); the second stage involved the use of previous efficiency scores to achieve regressions in order to determine the influencing factors. Based on these scores we performed a series of regressions with health, economic and social factors as independent variables. As input variables for the health system were considered COVID-19 cases, physicians, nurses, hospital beds, health expenditure, and for the output, COVID-19 deaths.

As can be seen from Tables 4 and 5, the efficiency of health systems is different for European countries and for the three stages. For the first period, namely first wave (January 1–June 15), the European states most affected by the pandemic were Belgium, Italy, Spain and the UK with rates below 0.5. These states had high rates of infection and disease transmission with a large number of infected people. It should be mentioned that these countries did not take strict lockdown measures from the beginning, which aggravated the intra-community transmission of the disease. Instead, the Eastern European countries, taking strict total lockdown measures, were not pressured by a large number of cases of illness. In this way, their health systems have not been put to major tests and thus explains the high efficiency in this phase. On the other hand, if we refer to what happened in the last part of 2020, more specifically in the second wave, we find that, this time, things have changed, in the sense that health systems from Eastern Europe highlighted their vulnerabilities related to medical infrastructure, medical personnel, coherent decisions, thus registering a very high number of deaths. This result comes against the background of taking accentuated relaxation measures in advance.

According to DEA results, the health systems of European countries were grouped into four categories of efficiency [38, 39], previously described. Through the regression analysis, it emerged that the determining factors for the large number of deaths caused by COVID-19 are those related to comobirdities, population age, and population density. For all elements that had a negative influence on efficiency, the most affected states (Belgium, Italy, Spain and the UK) had high values. The results obtained are in accordance with those obtained by [17, 18, 20]. Thus, the European countries have had different levels of efficiency and capabilities in rescuing patients infected with COVID-19. Although previous studies have shown that Western European states had more efficient medical systems before the pandemic, the reality of the first wave overturned this presumption. In the first wave, the Western states had an increased inefficiency in preventing deaths caused by COVID-19, while the Eastern ones, considered less prepared, had higher efficiency rates. The public health policies that have been applied in treating patients with COVID-19 have been more or less similar. The inefficiency of preventing COVID-19 deaths can lead to a focus on the analysis of public health policies promoted by countries. Throughout 2020 year, there have been countries that have experienced a steadily low inefficiency and countries that have managed to significantly reduce their inefficiency after a disastrous start. The current (in) efficiency of European medical systems in treating COVID-19 is closely dependent on the previous performance of health systems, and on their weaknesses or strengths. However, a number of inadequate public management strategies were promoted, especially at the beginning of the spread of the pandemic (the delay in taking strict measures of social distancing; the rapid installation of lockdown; the obligation to wear a mask).

For the relaxation phase (June 15–October 1), European states have gradually lifted previously imposed health restrictions. The situation changes radically from that of the first wave, in the sense that the Eastern states become ineffective in treating COVID-19. The lack of doctors and nurses, the poor financing of the health system, the faulty public policies in the field come to the fore with importance for them. Under these conditions, Bulgaria, Greece, Romania become the states with inefficient systems (< 0.35). These states stopped testing the population, allowed sick people to stay at home and thus infect other people; neglected to carry out the traceability of the disease among infected people. Western states, already hit hard in the first wave, have gained experience in treating the sick and their health systems have become more efficient. Western states have maintained and even continued a series of first-wave measures, massive testing of the population and isolation of the sick, wearing a mask, which has made the transmission of the disease low. The regression analysis shows that during this period government effectiveness is important, an indicator that has a positive influence on efficiency. Our results are similar to those obtained by [15, 23].

The second wave phase (October 1–December 31) shows a general deterioration in the efficiency of public health systems. However, this time too, the Eastern states are hardest hit by the disease (Hungary, Romania, Slovenia), but also a number of western states hard hit in the first wave (Belgium, Italy, UK). The other European states, even if they suffered a deterioration in efficiency, had a lower number of deaths caused by COVID-19. The effective states initiated a series of measures to counteract the disease such as massive population testing, social distancing, closing of the schools, the obligation to wear a mask, and finally, if these measures did not work, even temporary lockdown. In contrast, the Eastern states have not allocated sufficient resources to the health system, believing in most cases that the pandemic has passed. The governments of the respective states did not help the health system either, in the sense of promoting actions such as the obligation to wear a mask, social distance or closure of activities. The regression analysis shows that during this period the degree of education of the population was very important, which directly influenced the efficiency of the health system. Similar results were obtained by [13, 14, 16, 21].

In the second wave, the Western states coped much better with the pandemic than the Eastern ones. Strongly affected by the first wave, they learned from previous experience to manage COVID-19, allowing them to have a rapid medical response in the second wave. Public health management involved combining both sanitary actions (wearing a face mask, practicing social distancing, proper hand hygiene) and non-sanitary actions (closing public places, canceling public events, limiting transport capacity). Good quality public management, previously acquired, has led to the development of an appropriate behavior of the population (trust in health experts, information and warning system, technology involvement, leadership and participatory governance). Another important factor, lacking in most Eastern states, was the creation of a central department specialized in mobilizing the medical, financial and legislative resources needed to fight COVID-19 (procurement of medical supplies at the central level; channeling central resources to local departments in need; central online reporting system and databases; rapid deployment of medical staff from other sectors to intensive care units (ICUs); rapid transformation of hospital beds into ICUs). Western European countries, which were severely affected, in a first stage, have overtaken Eastern states in the second wave, due to better coordination between healthcare facilities; superior medical infrastructure; better transport systems; increased availability of medical experts, and increased public confidence in them.

Public health systems and especially their resources have intervened to rescue patients diagnosed with COVID-19. The public health system with its components has the essential role, from diagnosis, surveillance, disease control, in the various stages of the pandemic. Many European countries were not adequately prepared for a health crisis of this magnitude before, and they certainly are not at the moment either, thus showing the different levels of inefficiency.

## Conclusions and policy recommendations

Global pandemic caused by COVID-19 respiratory disease, triggered in December 2019, has required a reconfiguration of health systems, in order to provide the best responses in counteracting it. Since February 2020, the evolution of the disease has seen dramatic developments in European countries, many of them being hit dramatically by the first wave of the pandemic. They have taken various measures to slow down and limit the spread of the virus, making extensive efforts to control and mitigate it. Thus, the contribution of our research is given by the temporal analysis of the efficiency of the 31 European public health systems in time of COVID-19 pandemic, separately on the three phases corresponding to the period January 1–December 31, 2020.

After using the DEA methodology, the efficiency scores were estimated which show that only one country, Norway, was very efficient; the rest of the states have different degrees of inefficiency, higher or lower. The other European states recorded a series of scores including them in high efficiency (< 0.9; Cyprus, Denmark, Estonia, Finland, Iceland, Ireland, Luxembourg); average efficiency: (0.7–0.9; Austria, Croatia, Czech Republic, Latvia, Lithuania, Malta, Portugal, Slovakia, Slovenia, Switzerland); low efficiency: (< 0.7; Belgium, Bulgaria, France, Germany, Greece, Hungary, Italy, Netherlands, Poland, Romania, Spain, Sweden, UK).

The European countries analyzed in this study had different levels of inefficiency in saving the lives of patients with COVID-19. As we tried to explain in the study, although for some states (Italy, Belgium, Spain, UK), the literature states that although they have strong health systems, the sudden confrontation with a pandemic made them give way very easily. In Eastern European states, the COVID-19 pandemic only once again demonstrates the precarious state of their health systems. As our analysis shows, a simple comparison between European states is not enough to find the intrinsic causes of the high number of deaths. Public health measures, in principle similar, have caused some states to have different results in treating the disease, and others less so. Instead, most European countries have substantially increased the resources allocated to the healthcare system and especially to the treatment of COVID-19 patients. At the same time, practice has shown that the faster COVID-19 is diagnosed, the faster the chances of rescuing patients from the healthcare system increase. Under these conditions, in addition to the factors of the health system (doctors, beds, financing), there are a number of mechanisms specific to each state that must be taken into account in the analysis.

The high contagiousness of coronavirus requires prompt and coordinated measures, even if often they might not be in accordance with the wishes of a part of the population, for reasons related to education, culture, standard of living, historical past, expectations, etc. However, on the other hand, without their active participation in the implementation of government decisions the expected impact cannot be achieved, there is a division of society and even a spread of false news that this virus does not exist. Therefore, crisis management should be included in school curricula, in order to prepare future generations on how to act in the face of shocks of various kinds, how to prevent possible adverse effects, to be assumed as citizens of a state; regarding the decision-makers, it is urgently necessary to be offered by the specialized institutions some periodical training programs related to the adequate management of crises. In the long run, these actions can provide a bounce forward through adaptation and transformation and a favourable context for evidence-based decision making, taking into account the resource-needs relationship.

In some European countries, health systems need to go through a deep restructuring, which will lead to their strengthening, but also to a thorough assessment, both at the level of management and at the level of medical infrastructure. Without a clear inventory of inputs and outputs, the decisions will be inconsistent with the current reality and, therefore, they will fail to ensure national security; eloquent to this matter are the dozens of cases of fires caused in the intensive care units of some hospitals for infectious diseases in states that have not rationally invested in spending on health budgets. These situations have emerged as a consequence of overheating of the electricity installations, by connecting several artificial respirators and the impact on the population translates into a decline in confidence in the ability of governments to manage times of crisis. Thus, there is a need to regain this trust, through communication, transparency and openness to all elements likely to induce a rapid recovery. Also, restoring confidence in institutions, whether government or health, would be another key element in accepting the rules that must be taken to find a new pathway to overcome the pandemic.

In combating such a type of shock, immediate action is needed, the establishment of a recovery plan at all levels, the implementation of protocols agreed by the health system, which will determine individuals to trust more the medical environment. Thus, in the situation where, following the tests performed outside the hospital, cases of COVID-19 are detected, the respective persons should request specialized help, but not to resort to the application of their own treatments, as it has often been found that it happened. These people often prefer not to announce the directions of public health, especially in conditions where the symptoms are not aggravating, for reasons related to the exclusion of quarantine, rapidly spreading the virus in public space. Consequently, the awareness of the dangers arising from the adoption of such irresponsible behaviour must be induced to all citizens, by organizing constant information sessions, in which to explain how important it is to unite the efforts of all in eradicating the epidemiological situation. Then, humanitarian aid from more developed countries to those with lower possibilities to purchase sanitary materials (masks, disinfectants, oxygen devices) and vaccines has a special relevance in these turbulent times. Therefore, a successful transformation calls for responsibility, the involvement of all actors in society, institutional setups, clear policy and regulatory frameworks and absorptive and adaptive capacity.

Due to its nature and the uncertainties related to its evolution, the coronavirus pandemic must be treated with maximum responsibility by all actors in an economy, so as not to spread other more severe mutations, which would make it almost impossible to resist such shocks of health structures in less developed countries. In addition to the lack of financial, material and human resources, there is an obvious psychological crisis, which makes the system even more vulnerable. It should not be neglected that there are situations in which for fear of not contracting the disease in the hospital, the consultation of doctors is postponed, this leading to a chronicity of some diseases.

Although it is very difficult to measure accurately, the efficiency of health systems can be assessed in terms of the input-output relationship. Starting from this, overall, the results of our analysis show that there are a multitude of factors that influence the efficiency of health systems, which are not only associated with health issues, but also with economic, social, governmental aspects. Thus, it should be noted that the results of our study provide evidence to support the research hypothesis. In addition, it was highlighted that the variables that led to variations on the efficiency of European health systems were: for the first wave, significant in the efficiency of the health system are comobirdities, population age and population density; for relaxation period, a major role have government effectiveness and power distance, and for the second wave, relevant are education level and population density. For the first wave, the most efficient health systems were those in Austria, Denmark, Estonia, Finland, Iceland, and the most inefficient in Spain, the Netherlands, the UK, Italy and Belgium. For relaxation period, the most efficient health systems were those in Austria, Denmark, Estonia, Finland, Iceland, and the most inefficient were those in Poland, Sweden, Greece, Bulgaria and Romania. For the second wave, the most efficient health systems were those in Norway, Cyprus, Denmark, Iceland and Estonia, and the inefficient ones in Italy, Poland, Romania, Slovenia and Hungary.

Based on the experience of what the first year of the pandemic meant and starting from the results obtained, measures can be taken to prevent other waves at international level. The spread of the virus or of a new strains is gradual and, therefore, countries can better prepare their health systems so that the negative repercussions to be as small as possible. In addition, a certain degree of anticipation of possible implications arising from more severe coronavirus mutations may mean stronger shock resistance. As our analysis showed, a number of factors negatively influence efficiency (comobirdities, population age, and population density), while other factors positively influence the health systems (education and government effectiveness). Therefore, in a worldwide context, even if the elements that define health are very important, the aspects that refer to the cultural, socio-economic or institutional component should not be neglected, at least in the medium and long term. At the same time, the level of education and government effectiveness could shape the intensity of the health crisis. In a further study, it is challenging in building and visualizing bibliometric networks and seeing where our research is positioned in relation to these.

The health policies adopted have generated different results in European countries. Unable to change the size and structure of a short-term health care system, most of them have not improved their efficiency against COVID-19, but, in some cases, they have worsened it. Inefficient countries in the sample analyzed should learn from the effective ones some pandemic health management practices. At least for European countries, the efficiency of the public health sector is less determined by the endowment with medical resources, but rather by the correct use of available resources. Therefore, national public authorities in the event of pandemics should invest more in their proactive than reactive management. Immediate and adequate implementation of public health measures promoted by effective states should be followed by other states as well. Increased confidence in medical authorities and their proposed measures should be accepted by policy makers around the world, especially where the spread of the virus is very rapid. The synergy between the medical, political and population factors generates a global response to the fight against the pandemic. In addition to these considerations, public decision-makers should also take into account the complexity of the determinants (economic, social, technological, cultural) in the transmission of the virus, which is vital to cope with this period of pandemic.

All in all, we believe that in the fight against this epidemic, the approach must be one assumed by both parties: on the one hand, by the population, by adapting to a new way of life (traffic restrictions, working from home – where possible) and compliance with safety rules, and on the other hand, by authorities, be they local, regional or national; the latter should incorporate in policies and strategies to combat this crisis, the experiences acquired from previous shocks since, at EU level, a transformational change is needed to involve the proactive participation of all actors in finding the best solutions to ensure the premises for the exit of states from the crisis.

## Data Availability

The data can be extracted from Worldometer, World Health Organization (WHO) and World Bank databases. However, the data used in the analysis will be available upon request.

## Appendix 1. Stringency index – comparative approach between the old and the new EU member states

*Source:* Oxford Coronavirus Government Response Tracker (OxCGRT)

**COVID-19: cases and deaths in first wave, relaxation and second wave.**

## Abbreviations

DEA: Data Envelopment Analysis
WHO: World Health Organization
Max: Maximum
Min: Minimum
OECD: Organisation for Economic Co-operation and Development
GDP: Gross Domestic Product
DMU: Decision making unit
